# The other-race effect of pupil contagion in infancy

**DOI:** 10.1038/s41598-024-59937-0

**Published:** 2024-04-24

**Authors:** Yuki Tsuji, So Kanazawa, Masami K. Yamaguchi

**Affiliations:** 1 Organization for the Strategic Coordination of Research and Intellectual Properties, Meiji University, 1-1-1 Higashimita, Tama-ku, Kawasaki, Kanagawa 214-8571 Japan; 2 Department of Psychology, Japan Women’s University, 2-8-1 Mejirodai, Bunkyo-ku, Tokyo 112-8681 Japan; 3 Department of Psychology, Chuo University, 742-1 Higashi-Nakano, Hachioji, Tokyo 192-0393 Japan

**Keywords:** Human behaviour, Cognitive neuroscience

## Abstract

Pupil contagion refers to the observer’s pupil-diameter changes in response to changes in the pupil diameter of others. Recent studies on the other-race effect on pupil contagion have mainly focused on using eye region images as stimuli, revealing the effect in adults but not in infants. To address this research gap, the current study used whole-face images as stimuli to assess the pupil-diameter response of 5–6-month-old and 7–8-month-old infants to changes in the pupil-diameter of both upright and inverted unfamiliar-race faces. The study initially hypothesized that there would be no pupil contagion in either upright or inverted unfamiliar-race faces, based on our previous finding of pupil contagion occurring only in familiar-race faces among 5–6-month-old infants. Notably, the current results indicated that 5–6-month-old infants exhibited pupil contagion in both upright and inverted unfamiliar-race faces, while 7–8-month-old infants showed this effect only in upright unfamiliar-race faces. These results demonstrate that the face inversion effect of pupil contagion does not occur in 5–6-month-old infants, thereby suggesting the presence of the other-race effect in pupil contagion among this age group. Overall, this study provides the first evidence of the other-race effect on infants’ pupil contagion using face stimuli.

## Introduction

Pupil contagion is the involuntary change in the pupil diameter of an observer in response to another person’s pupil size. Recent studies have demonstrated that pupil contagion occurs in chimpanzees^1^, adults^2–6^, and infants^7–13^. Accumulating evidence indicates that pupil contagion is a social aspect extending beyond physiological reactions^1–6,10–13^. One of the social aspects of adult pupil contagion has been reported that pupil contagion was induced by other’s pupil dilation which could indicate emotional arousal^14,15^. Additionally, as one of the social aspects of infant’s pupil contagion, a recent study reported an emotional response^11^. Tsuji and colleagues’ study^11^ investigated whether an emotional response would occur in pupil contagion by using skin conductance response (SCR) in 5- and 6-month-old infants. The emotional responses to pupil diameter change (dilating/constricting) between the face and eye region were compared using pupil diameter response and SCR. The results revealed a significant increase in the SCR when participants looked at dilating pupils of face stimuli compared to constricted pupils of face stimuli, but this increase did not occur when they looked at eye region stimuli. This suggests that the face plays a crucial role in inducing emotional response, one of the social aspects, in an infant’s pupil contagion.

Interestingly, the social aspect of adult pupil contagion has been reported to depend on the social group to which the observed individuals belong (i.e., the in-group). Kret et al.^3,4^ showed that adult pupil contagion was correlated with trust within the one’s in-group partners as dilating pupils. They measured participants’ pupil size while they played incentivized trust games with virtual partners whose pupils were dilated, static, or constricted. Eyes from the one’s in-group (Dutch men and women) and out-group (Japanese men and women) were used. Their results showed that the participants synchronized their pupil size with that of the dilated pupils. This notion only applies to eye regions of the one’s in-group. Thus, the other-race effect was found in adult pupil contagion when utilizing eye-region images as stimuli.

Currently, there is no evidence of the other-race effect on infant pupil contagion. A study using eye-region images as stimuli investigated this effect in 9–14-month-old infants^9^, however, the other-race effect was not observed. Adult studies^3,4^ using the same eye-region images as stimuli demonstrated the other-race effect. Interestingly, infant face studies have reported that the other-race effect emerged by 9 months of age^16,17^. This suggests that they may require more information than just the eye region to exhibit this effect. We assumed that the face as a stimulus would be more effective in inducing the other-race effect of pupil contagion in infants due to the additional information contained in facial color and shape.

In our previous study^10^, we examined the effect of the face on infants’ pupil contagion by measuring the pupil diameter of 5–6-month-old infants in response to changes in pupil size (dilation/constriction) in both upright and inverted familiar-race faces. The results showed that infants displayed a greater pupil diameter response to pupil dilation than to pupil constriction in upright faces, whereas, in inverted faces, the pupil diameter response between dilating and contracting pupils did not differ significantly. These results indicate that pupil contagion occurred in upright faces but not in inverted faces, suggesting the presence of the face-inversion effect of pupil contagion in familiar-race faces. In general, the face-inversion effect is regarded as a hallmark of face processing^18,19^, and our results suggest that the face does indeed influence infants’ pupil contagion in familiar-race faces.

However, this face-inversion effect of pupil contagion to familiar-race faces with extensive perceptual experience does not necessarily reflect solely face processing. The inversion effect, traditionally associated with face processing, may also reflect perceptual expertise or specialization. Gong et al.^20^ suggested that the effect was indicative of specific processing in face recognition or expertise. This is supported by Diamond and Carey’s study^21^, which found that dog experts, like face experts, were also affected by the inversion effect. Cashon and Holt^22^ underscored this by showing that the developmental origins of the inversion effect begin in infancy, suggesting a specialized system for upright faces. Belle et al.^23^ provided evidence that the inversion effect impairs holistic perception, which is a key component of expertise in face recognition. These studies collectively suggest that the inversion effect is not solely a product of face processing, but also reflects perceptual expertise or specialization. On the other hand, if perceptual experience with unfamiliar-race faces is limited, the inversion effects observed on unfamiliar-race faces may reflect aspects of facial processing. This would also be established in the face-inversion effect of pupil contagion for unfamiliar-race faces.

Many infant face studies have reported the other-race effect of the face, which refers to the impaired identification and recognition of unfamiliar-race faces relative to familiar-race faces. The other-race effect of the face has been widely observed across various races and ethnicities^24^, including in monoracial and multiracial societies^25^. In addition, the other-race effect has been shown to occur in humans from infancy^16,17,26–28^, childhood^29^, and adolescence to adulthood^25^. Given that the other-race effect has been well-documented in existing literature, it is likely that the other-race effect would also be demonstrated in pupil contagion using face images.

Several studies have reported infants’ pupil contagion. Fawcett et al.^8^ demonstrated that schematic images of eye-regions induced pupil contagion in 6- and 9-month-old infants. Pupil contagion was observed to be induced by pupils^7,13^ or pupil changing^9,11,12^ in familiar-race eye regions images in infants at various ages (4- and 6-month-old^7,13^; 5–6 month-old^11^; 10 month-old^12^; 6-, 12-, and 18 month-old^9^). Pupil changing in familiar-race face images induced pupil contagion in 5–6 month-old infants^10^. These findings suggest that pupil contagion can be induced by pupils in familiar-race eye regions or face images in infants older than 4 months. Previous infants’ pupil mimicry study reported that eyes of unfamiliar-race induced pupil mimicry in older infants^9^. They measured 9–14 month-old infants’ and their parents’ pupil diameter response to own-race and other-race eye regions with static, constricting, or dilating pupils. The results showed that both infants and parents responded more to dilating pupils than to static and constricting pupils, regardless of the race. This indicates that eyes images did not induce the other-race effect in pupil mimicry. However, to the best of our knowledge, no studies using unfamiliar-race face images as stimuli have been reported. Infant face studies have shown the other-race effect. Our previous study reported pupil contagion depends on the facial context. Therefore, we predicted that facial context would play an important role in inducing the other-race effect in infants’ pupil contagion. Demonstrating the other-race effect of infants’ pupil contagion within a facial context would provide evidence that early environmental experiences influence physiological interactions, which could assist in understanding the mechanisms underlying the social aspects of pupil contagion.

The present study aimed to demonstrate the other-race effect of pupil contagion in infancy. A previous study^9^ reported that pupil contagion occurred in infants aged 9–14 months using unfamiliar-race eyes region images. However, it has not been clear whether infants’ pupil contagion occurred using the unfamiliar-race face images. We investigated whether 5–8 month-old infants would exhibit pupil contagion in response to unfamiliar-race faces. The Experiment 1 involved measuring the 5–6 month-old infants’ pupil diameter response to changes in pupil size (dilation/constriction) of both upright and inverted unfamiliar-race faces. When pupil contagion occurs, the pupil diameter response to dilating pupils is larger than the pupil diameter response to constricting pupils. Our previous study, which used familiar-race face images as stimuli, demonstrated that 5–6 month-old infants’ pupil responses were asymmetrical in upright faces but not in inverted faces^10^. This suggests that pupil contagion disappeared in inverted faces because 5- to 6 month-old infants are still immature in their ability of face processing and have difficulty processing inverted faces as faces. Based on these findings, it was hypothesized that pupil contagion would not occur in unfamiliar-race faces unfamiliar-race faces, unlike in familiar-race faces^10^. Therefore, it was predicted that the pupil diameter response of 5–6 month-old infants to dilating pupils and contracting pupils of unfamiliar-race faces would not differ in upright and inverted faces. Pupil contagion may improve as infants develop their ability of face processing since our previous study indicated that pupil contagion was influenced by face. Moreover, infant face studies have reported that the other-race effect emerged by 6 months of age and was present at 9 months of age^16,17^. We hypothesized that the other-race effect might occur in older infants’ pupil contagion to pupil change of unfamiliar-race faces*.* Thus, we investigated whether 7–8 month-old infants would exhibit pupil contagion in response to unfamiliar-race faces in Experiment 2.

## Results

### Experiment1

To determine the occurrence of the other-race effect on pupil contagion, we examined 5–6 month-old infants’ pupil response to pupil-change directions (dilating/constricting) of upright and inverted unfamiliar-race faces.

**Figure 1:**
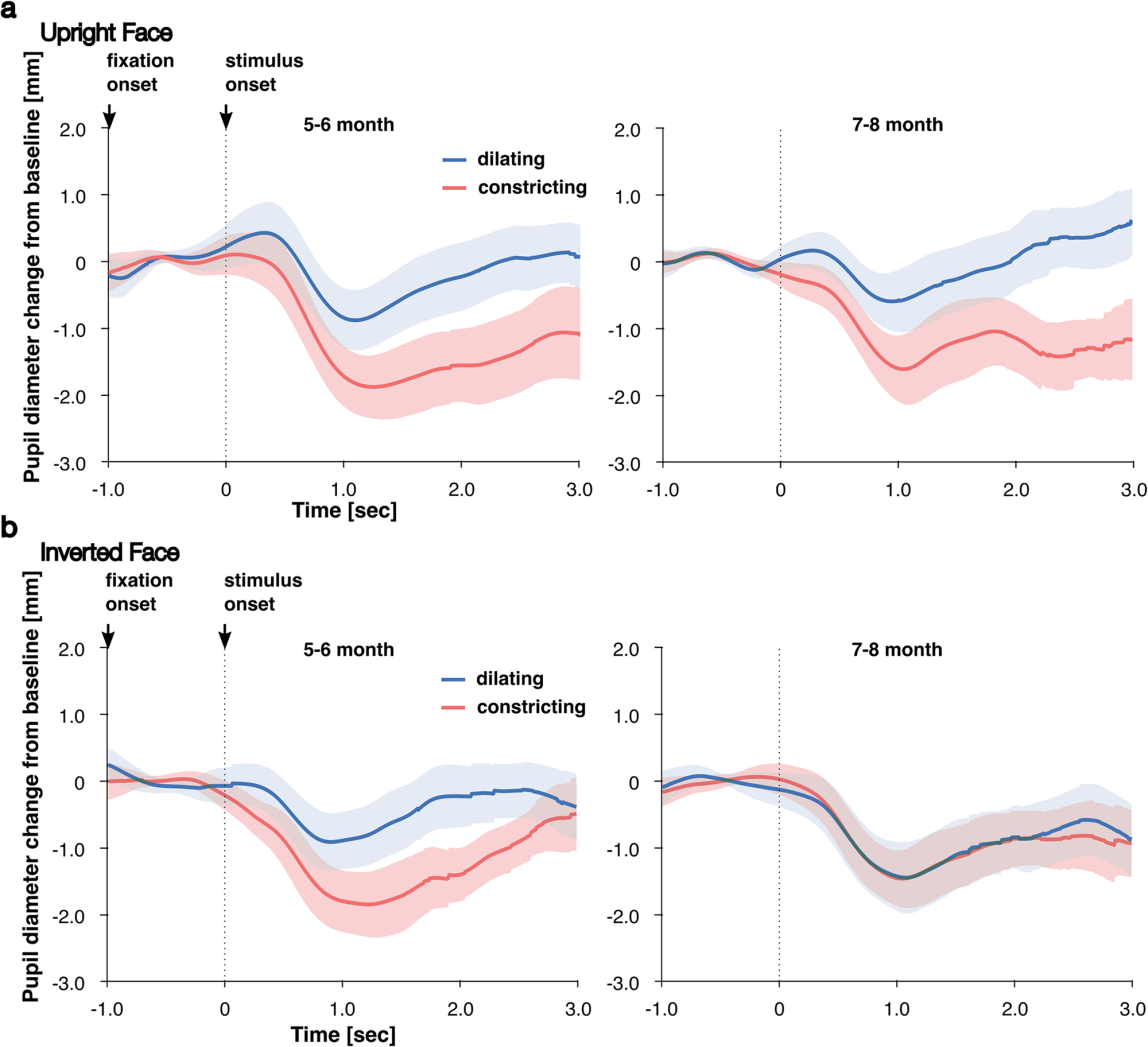
Infants’ pupil-diameter response to pupil changing (dilating/constricting) of unfamiliar-race faces.﻿ The waveforms represent the time course of pupil-diameter response to dilating (blue lines) and constricting (red lines) pupils of upright (**a**: top panels) and inverted faces (**b**: bottom panels). The left panels represent 5–6-month-old infants’ pupil-diameter response (Exp.1). Right panels represent 7–8-month-old infants’ pupil-diameter response (Exp.2). Shaded regions indicate 95% confidence intervals.

The mean pupil diameters were evaluated via a two-way ANOVA with face orientation (upright or inverted) as a between-subject factor and pupil-change direction (dilating or constricting) as a within-subject factor. The two-way ANOVA revealed a significant main effect of pupil-change direction (F[1, 48] = 91, p < 0.01, η^2^ = 0.65). Thus, the mean pupil diameter in response to dilating pupils was larger than that in response to constricting pupils. The main effect of face orientation (F[1, 48] = 0.15, p = 0.70, η^2^ = 0.0031) and the interaction between face orientation and pupil-change direction (F[1, 48] = 1.4, p = 0.24, η^2^ = 0.029) were not significant. These results showed that pupil-diameter responses to dilating pupils were larger than pupil-diameter responses to constricting pupils (i.e., pupil contagion occurred for unfamiliar-race faces, Fig. 1: left panels). This notion does not support our hypothesis that the pupil-diameter responses between dilating and contracting pupils would not differ. The outcomes of Experiment 1 confirmed that pupil contagion to pupil change occurred for upright and inverted unfamiliar-race faces.

### Experiment 2

We investigated whether older infants’ pupil contagion to pupil change would occur in unfamiliar-race faces because pupil contagion occurred in Experiment 1. We examined 7–8-month-old infants’ pupil response to the pupil-change direction (dilating/constricting) of upright and inverted unfamiliar-race faces to determine whether other race effects on pupil contagion would occur.

The mean pupil diameters were assessed using a two-way ANOVA with face orientation (upright or inverted) as a between-subject factor and pupil-change direction (dilating or constricting) as a within-subject factor. The two-way ANOVA revealed a significant main effect of pupil-change direction (F[1, 48] = 64, p < 0.01, η^2^ = 0.57) and a significant interaction between face orientation and pupil-change direction (F[1, 48] = 56, p < 0.01, η^2^ = 0.53). The main effect of face orientation (F[1, 48] = 1.4, p = 0.24, η^2^ = 0.029) was not significant. Post hoc analysis (simple main effect tests) of the interaction revealed that the mean pupil diameter in response to dilating pupils was larger only in the upright face than that in response to constricting pupils (upright face: F[1, 24] = 100, p < 0.01, η^2^ = 0.81; inverted face: F[1, 24] = 0.16, p = 0.69, η^2^ = 0.0066). The mean pupil diameter in response to the dilating pupils was also greater when the face orientation was upright compared to when it was inverted (F[1,48] = 13.0, p < 0.01, η^2^ = 0.18). These findings showed that in the upright unfamiliar-race faces, the pupil-diameter response to dilating pupils was larger than the pupil-diameter response to constricting pupils, but in the inverted unfamiliar-race faces, the pupil-diameter response between dilating and contracting pupils did not differ (Fig. 1: right panels). This notion does not support our hypothesis that the pupil-diameter response between dilated and constricted pupils would not differ. Unlike Experiment 1, the results of Experiment 2 denote that 7–8-month-old infants’ pupil contagion to pupil change occurred for the upright face, but pupil contagion did not occur for the inverted face. Accordingly, the face inversion effect occurred in 7–8-month-old infants’ pupil contagion.

### Further analysis (Experiment 1 & 2 ANOVA)

The results of Experiments 1 and 2 did not support our hypothesis that pupil contagion would not occur in unfamiliar-race faces among 5–6-month-old and 7–8-month-old infants. However, pupil responses to pupil changes (dilating/constricting) in inverted unfamiliar-race faces differed between 5–6-month-old and 7–8-month-old infants. On the upright unfamiliar-race face, 5–6-month-old and 7–8-month-old infants’ pupil-diameter responses to dilating pupils were larger than pupil-diameter responses to constricting pupils. In the inverted unfamiliar-race face, 5–6-month-old infants’ diameter response to dilating pupils was larger than the pupil-diameter response to constricting pupils, while 7–8-month-old infants’ diameter responses between dilating pupils and constricting pupils did not differ. To investigate this difference, we conducted a three-way ANOVA, including age groups (5–6-month-olds and 7–8-month-olds) as between-subjects factors. For this further analysis, we adjusted for multiple comparisons using Bonferroni correction (n = 3).

The mean pupil diameters were analyzed using a three-way ANOVA with face orientation (upright face or inverted face) and age group (5–6-month-old or 7–8-month-old) as between-subject factors and pupil-change direction (dilating or constricting) as within-subject factors (10, 30). The three-way ANOVA revealed a significant main effect of pupil-change direction (F[1, 96] = 121, p < 0.01 after Bonferroni correction, η^2^ = 0.558), a significant interaction between face orientation and pupil-change direction (F[1, 96] = 29.2, p < 0.01 after Bonferroni correction, η^2^ = 0.233), and a three-way interaction between face orientation, age group, and pupil-change direction (F[1, 96] = 7.94, p < 0.05 after Bonferroni correction, η^2^ = 0.0764). Thus, follow-up ANOVAs (simple main effect tests) were conducted as post hoc analyses, and these tests revealed that there were significant simple interactions between face orientation and pupil-change direction among 7–8-month-old (F[1, 48] = 56.3, p < 0.01 after Bonferroni correction, η^2^ = 0.540) and between age group and pupil-change direction in the inverted face (F[1, 48] = 11.0, p < 0.01 after Bonferroni correction, η^2^ = 0.196). Further, in the 5–6-month-old group, the mean pupil diameter for the dilating pupil was larger than that of the constricting pupil in the upright (F[1, 24] = 55.6, p < 0.01 after Bonferroni correction, η^2^ = 0.699) and inverted face (F[1, 24] = 16.9, p < 0.01 after Bonferroni correction, η^2^ = 0.413), but in the 7–8-month-olds, the mean pupil diameter of the dilating pupil was larger than that of the constricting pupil only in the upright face (F[1, 24] = 103, p < 0.01, η^2^ = 0.810) as opposed to the inverted face (F[1, 192] = 0. 16, p = 0.693, η^2^ = 0.00661). These results signify that the 5–6-month-old infants’ pupil contagion occurred in the upright and inverted face orientations, whereas among the 7–8-month-old infants, it occurred only in the upright face. In the 7–8-month-old group, the mean pupil diameter of the dilating pupil of the upright face was larger than that of the inverted face (F[1, 48] = 10.2, p < 0.01 after Bonferroni correction, η^2^ = 0.175). Hence, the 7–8-month-old infants’ pupil contagion to dilating pupils occurred only on the upright face. Altogether, in upright unfamiliar-race faces, pupil contagion occurred in 5–6-month-old and 7–8-month-old infants, and in inverted unfamiliar-race faces, pupil contagion occurred only in 5–6-month-old infants. These results of further analysis were consistent with the ANOVA results of Experiment 1 and those of Experiment 2. Accordingly, the face inversion effect occurred in 7–8-month-old infants’ pupil contagion to unfamiliar-race faces.

Additionally, we also applied a Generalized linear mixed model (GLMM) to analyze the pupil data^30–32^. The GLMM was applied with pupil change direction (dilating or constricting), and face orientation (upright or inverted), age (5–6-month-old or 7–8-month-old), and all their interactions as fixed effects, and with trial and individual differences as random effects, to analyze infants’ pupil data (model selection detail see Supplementary Material 1). The GLMM revealed a significant effect (pupil change direction: b = 0.0847, SE = 0.0197, t = 4.30, p < 0.001), a significant interaction between age group and pupil-change direction (b = − 0.0714, SE = 0.0254, t = -2.81, p < 0.001), and a three-way interaction between face orientation, age group, and pupil-change direction (b = 0.0883, SE = 0.0299, t = 2.95, p < 0.001), that is, the GLMM result consisted with the ANOVA results.

## Discussion

This study investigated whether pupil contagion to pupil change occurs in the faces of unfamiliar-race. In Experiment 1, to investigate this point, we measured 5–6-month-old infants’ pupil-diameter response to the pupil-change direction (dilating/constricting) of upright and inverted unfamiliar-race faces. We hypothesized that if pupil contagion did not occur in unfamiliar-race faces, the pupil-diameter response between dilating and contracting pupils would not differ. The results of Experiment 1 showed that 5–6-month-old infants’ pupil-diameter response to dilating pupils was larger than the pupil-diameter response to constricting pupils of the upright and inverted unfamiliar-race faces. Therefore, 5–6-month-old infants’ pupil contagion to pupil changing occurred on both unfamiliar-race faces. However, this finding did not support our hypotheses. As pupil contagion to unfamiliar-race faces occurred in Experiment 1, we further investigated in Experiment 2 whether older infants might show pupil contagion to unfamiliar-race faces. The results highlighted that 7–8-month-old infants’ diameter response to dilating pupils was larger than the pupil-diameter response to constricting pupils of the upright unfamiliar-race faces. Hence, 7–8-month-old infants’ pupil contagion to pupil changing had occurred. The outcomes of Experiments 1 and 2 did not support our hypothesis. Additionally, 7–8-month-old infants’ pupil-diameter response between dilating and constricting pupils did not differ in the inverted unfamiliar-race face. To explain the difference in pupil-diameter response to the inverted unfamiliar-race face between 5–6-month-old and 7–8-month-old, we conducted a three-way ANOVA, including age groups (5–6-month-old and 7–8-month-old) as a between-subject factor. The findings outlined that in the upright unfamiliar-race face, 5–6-month-old and 7–8-month-old infants’ diameter response to dilating pupils was larger than the pupil-diameter response to constricting pupils (i.e., pupil contagion had occurred). Regarding inverted unfamiliar-race faces, 5–6-month-old infants’ diameter response to dilating pupils was larger than the pupil-diameter response to constricting pupils, but 7–8-month-old infants’ diameter response between dilating and constricting pupils did not differ. Thus, 5–6-month-old infants’ pupil contagion occurred in response to inverted unfamiliar-race faces, but 7–8-month-old infants’ pupil contagion did not occur in response to inverted unfamiliar-race faces.

In this study, using faces of unfamiliar-race, 5–6-month-old infants’ pupil contagion occurred in upright and inverted faces. This suggests that the face-inversion effect did not occur in 5–6-month-old infants’ pupil contagion to unfamiliar-race faces, given that task performance is generally worse for inverted faces in the face-inversion effect. Conversely, our previous study^10^ of pupil contagion using familiar-race faces showed that 5–6-month-old infants’ pupil contagion did not occur in the inverted face but in the upright face. This denotes that the face-inversion effect occurred in 5–6-month-old infants’ pupil contagion to familiar-race faces. The face-inversion effect is generally considered a hallmark of face processing^18,19^. These findings indicate that face processing might be involved in pupil contagion to familiar-race faces but not to unfamiliar-race faces in 5–6-month-old infants. We assume that the other-race effect occurred in 5–6-month-old infants’ pupil contagion.

The current study found that 7–8-month-old infants demonstrated pupil contagion to unfamiliar-race faces when the faces were upright, but not when they were inverted. This means that the face-inversion effect occurred in 7–8-month-old infants’ pupil contagion to unfamiliar-race faces. However, our previous study^10^ found that 5–6-month-old infants exhibited the face inversion effect of pupil contagion to familiar-race faces. These result indicate that face processing was involved in pupil contagion to familiar-race faces among 5–6-month-old infants and unfamiliar-race faces among 7–8-month-old infants, given that the face-inversion effect is generally considered a hallmark of face processing^18,19^. Additionally, pupil contagion to familiar-race faces or eye regions is documented in infants aged 4 to 18 month (4- and 6-month old^7,13^; 5–6-month-old^10,11^; 6-, 12- and 18-month old^9^; 10-month old^12^). Considering previous studies of infants’ pupil contagion using familiar-race eye regions, using familiar-race face pupil contagion might also occur in 5–6- and 7–8-month-old infants. In summary, the results of this study and our previous study revealed that face processing was not involved in pupil contagion to unfamiliar-race faces in 5–6-month-old infants, however, it was involved in pupil contagion to unfamiliar-race faces in 7–8-month-old infants and to familiar-race faces in 5–6-month-old infants. We found the age difference in the other-race effect of pupil contagion.

The other-race effect shown by our study differ from the traditional view. In traditional views, the other-race effect is observed in younger infants demonstrating perceptual discrimination or expertise in processing faces of both unfamiliar and familiar races. Our study found that younger (5–6-month) infants showed the face-inversion effect of pupil contagion only for familiar races, older (7–8-month) infants showed the face-inversion effect of pupil contagion for familiar- and unfamiliar-race faces. This difference in the face-inversion effect of pupil contagion between familiar- and unfamiliar-race faces could be referred to as the other-race effect of pupil contagion.

Our study investigated the other-race effect in infants’ pupil contagion, similar to the study by Aktar et al.^9^ except that the stimuli presented were either eyes or faces, but with different results. Aktar and collegues^9^ investigated the other-race effect in 9–14-month-old infants’ pupil mimicry using eye region images as stimuli, however, they did not find the effect. They measured infants’ and their parents’ pupil diameter response to own-race and other-race eye regions with static, constricting, or dilating pupils. The results showed that both infants and parents responded more to dilating pupils than to static and constricting pupils, regardless of the race of the individual. This indicates that the other-race effect was not observed in the pupil mimicry of both 9–14-month-old infants and their parents in response to the eye region. Our study investigated the other-race effect in 5–6-month-old and 7–8-month-old infants’ pupil contagion by using face images as stimuli. The study identified a significant other-race effect in 5–6-month-old infants’ pupil contagion. The study measured the infants’ pupil diameter response to dilating or constricting pupils in both upright or inverted unfamiliar-race faces. As indicated above, the results showed that 5–6-month-old infants did not exhibit the face-inversion effect of pupil contagion for unfamiliar-race faces, unlike 7–8-month-old infants. In contrast, our previous study demonstrated that 5–6-month-old infants did exhibit the face-inversion effect of pupil contagion for familiar-race faces. The difference in the face-inversion effect of pupil contagion between the familiar-race and the unfamiliar-race faces is likely due to the other-race effect of the face. The current study is the first to demonstrate the other-race effect of the face on 5–6-month-old infants’ pupil contagion by using face images as stimuli.

The differences in results between our study and Aktar’s study^9^ may be attributed to differences in stimuli and the information contained in each stimulus. Our study demonstrated that using whole-face images as stimuli induced the other-race effect of pupil contagion in 5–6 month-old infants, while Aktar’s study^9^ reported that using only the eye region images as stimuli did not induce the other-race effect of pupil contagion in 9–14-month-old infants. Our face image stimuli included multiple morphological features, such as eyes, nose, and mouth, whereas their stimuli only included the single morphological feature of eyes. Since our study’s whole-face stimuli included more morphological features than their eyes region stimuli, it is possible that our stimuli containing greater facial information may have induced the other-race effect of pupil contagion.

We speculate that the other-race effect of pupil contagion in 5–6-month-old infants may have been affected by face processing. Both our and Aktar et al.’s studies^9^ provided similar results, where the other-race effect of pupil contagion did not occur despite using different stimuli. However, our results indicate that the other-race effect of the face was present. This study investigated the face-inversion effect on pupil contagion and compared the pupil contagion between upright and inverted faces. The results indicated that in upright faces, pupil contagion occurred regardless of the race of the face, in 5–6-month-old infants. However, in inverted faces, pupil contagion did not occur for familiar-race faces, but it did for unfamiliar-race faces. This suggests that the face-inversion effect of pupil contagion did not occur in unfamiliar-race faces and highlights the other-race effect of pupil contagion, which could be influenced by face processing. Previous studies on face recognition^27^ and identification^28^ have reported the other-race effect in infants, with better performance observed for familiar-race faces than unfamiliar-race faces^26^. The other-race effect of the face has been observed as early as three months of age^26,27^ and as late as six months of age^28^, and the other-race effect of pupil contagion is found almost the same age. In the current study, the other-race effect of pupil contagion disappeared at 7–8 months, unlike the acquisition of the other-race effect of the face.

The possibility cannot be excluded that the inversion effect of pupil contagion to unfamiliar-race faces did not observe in 5–6-month-old infants due to a lack of perceptual experience. Given that the inversion effect may also be induced by perceptual specialization, this absence may not be due to immature face processing but rather to a lack of perceptual experience for unfamiliar objects. However, despite the lack of exposure to unfamiliar-race faces (as self-reported by parents), the face-inversion effect of pupil contagion to unfamiliar-race faces was observed in 7–8-month-old infants. That is, the inversion effect of pupil contagion to unfamiliar race faces was observed even though perceptual specialization to unfamiliar race faces did not occur. This suggests an improvement of processing unfamiliar-race faces in 7–8-month-old infants.

Integrating the above, the face-inversion effect of pupil contagion occurred in familiar-race faces disappeared in unfamiliar-race faces, which would be the other-race effect of pupil contagion in infancy. Furthermore, the other-race effect of pupil contagion disappears with development unlike traditional the other-race effect. This suggests an improvement of processing unfamiliar-race faces because the inversion effect of pupil contagion to unfamiliar race faces was observed even though perceptual specialization to unfamiliar race faces did not occur. To conclude, the other-race effect of pupil contagion would reflect the ability of face-processing in infancy.

Finally, we speculate that two factors contributed to the other-race effect on pupil contagion in the current study. One possible explanation for this other-race effect is the luminance difference between Japanese and White. Although, we adjusted the mean luminance of all face images in this experiment to closely align with those in our previous study (211 cd/m^2^ vs. 212 cd/m^2^), the luminance of the Japanese-eyes region (204 cd/m^2^ ± 4.11 [SD]) differed from that of the White-eyes region (190 cd/m^2^ ± 4.78 [SD]). The difference in the luminance of eye region between Japanese and White is due to unique facial topographies of each face. White face has prominent brows ridge and high bridge of the nose that cast dark shade on the eye region, while Japanese flat faces cast light shade on the eye region. If this luminance difference of the eye region between races influences pupil contagion, different pupil contagion would be observed between races regardless face orientation. However, our results showed that 5–6-month infant’s pupil contagion did not differ between races in the upright faces, while differ between races in the inverted face. The difference in pupil contagion between races for inverted faces is not due to luminance, since the luminance is the same for upright and inverted faces. This finding suggests that the difference in luminance between races might not influence infant’s pupil contagion at this age. Aktar et al.^9^ have also showed that the difference in luminance of eye images between races did not influence pupil contagion, as infants’ pupil contagion did not differ between races. Thus, infant’s pupil contagion might not be influenced by such low-level difference in stimuli.

Another possibility is that the current study’s other-race effect on pupil contagion is due to the minimal exposure to unfamiliar-race faces. To assess infants’ experiences with unfamiliar-race faces, we asked parents of participants about participants’ experience with White female faces and all infants had little or no exposure to them in the current study. Moreover, we investigated demographic data on foreign nationals residing in Tokyo, where the study participants were located. The results indicate that foreign children are enrolled in many preschools (81.5%, as of 2016), and most foreign nationals are from Asia (86.3%, as of 2013). These data suggest that little or no experience with White female faces contributed to the occurrence of the other-race effect of pupil contagion.

### Limitations

This research is based on comparison with previous research. Thus, the findings of our study must be seen in the light of the following limitations: First, replication of familiar-race face processing did not examine in this current study. We have not investigated 7–8-month infants’ pupil contagion in upright or inverted familiar-race faces. It is unclear whether the face-inversion effect of pupil contagion observed in 5–6-month infants replicated in 7–8-month infants. Complementing the lack of data will further our understanding of the other-race effect of infants’ pupil contagion. Second, there is a statistical limitation that should be considered. We designated the face orientation (upright or inverted) as the between-subjects factor in our experiment. The comparison of the conditions of the upright and inverted face reduces the statistical power of the comparison and thus the generalizability of the data. To solve this statistical limitation, all conditions, face orientation (upright or inverted), pupil changing direction(dilation or constriction) and age (5–6-month or 7–8-month), should have been implemented within-subject factors. However, if all factors were within-subject factors, infants would have difficulty completing the experiment because of the length of the experiment. Further research is needed to examine the generalizability of the data.

## Materials and methods

The methods and stimuli used in Experiment 1 and 2 were the same, except for the participants.

### Participants

*Experiment 1*: Overall, 50 5–6-month-old East Asian infants (32 females and 18 males born and raised in Japan; age range: 138–195 days; mean age: 169.4 days) participated in Experiment 1 consisting of factors of face orientation (between-subject factor: upright face versus inverted face) and pupil-change direction (within-subject factor: dilating versus constricting). Participants were randomly assigned to one of the two face-oriented conditions. An additional 24 infants participated but were not included in the final analysis because of fussiness (n = 5, 5-month-old and n = 4, 6-month-old infants), machine trouble (i.e., inability to calibrate gaze; n = 4, 5-month-old and n = 3, 6-month-old infants), or insufficient data (n = 4, 5-month-old and n = 4, 6-month-old infants; at least 50% or more sampled pupil diameter data per pupil-change direction were required for inclusion). Experiment 2: Overall, 50 7–8-month-old East Asian infants (26 females and 24 males born and raised in Japan; age range: 196–254 days; mean age: 226.0 days) participated in *Experiment 2* consisting of factors of face orientation (between-subject factor: upright face versus inverted face) and pupil-change direction (within-subject factor: dilating versus constricting). Participants were randomly assigned to one of the two face-oriented conditions. An additional 17 infants participated but were not included in the final analysis because of fussiness (n = 4, 7-month-old and n = 2, 8-month-old infants), machine trouble (i.e., inability to calibrate gaze; n = 2, 7-month-old and n = 2, 8-month-old infants), or insufficient data (n = 3, 7-month-old and n = 4, 8-month-old infants; at least 50% or more sampled pupil-diameter data per pupil-change direction were required for inclusion).

Parents were asked about the frequency of infants’ exposure to White female faces to assess their viewing experiences, and all infants had almost none. Infants were recruited through newspaper advertisements. All infants were full-term at birth and healthy at the time of the experiment. Written informed consent was obtained from the parents of all participants. The study protocol was approved by the ethics committee of Chuo University (approval number: 2020-39), and the study was conducted in accordance with the principles and guidelines of the Declaration of Helsinki. Parents provided written informed consent for their children’s participation and the publication of the results in an online open-access publication.

### Stimuli

In total, six facial images of White females were used as unfamiliar-race images. As infants prefer female faces, we solely employed female faces to draw their attention to the stimuli. The images were created using the following procedure. First, symmetrical eye regions were created on each side to avoid excessive attention. To create faces with symmetrical eye regions, we extracted the side of the face containing over 80% of the visible iris, which was vertically flipped and merged into the entire face. Each facial image was cropped to an oval shape (15.7° × 18.5°). Furthermore, we gradually changed the color from face to background (R, G, B = 171) to reduce the contrast at the boundaries between the face and background. The mean luminance (212 cd/m2 ± 0.48 [SD]) of the entire face was made uniform, except for the irises and pupils. The average overall luminance of the images was 211 cd/m2 ± 0.036 (SD), and the eyes were then filled with new irises, which were gray (15 cd/m2), and an artificial pupil was added using GIMP vers. 2.10.4. The distance between the left and right pupils on each face was 5.8 cm.

The static artificial pupil diameter (5.0 mm) was presented for 0.5 s. Thereafter, in the constricting condition, the artificial pupil diameter was constricted by 60% (from 5.0 mm to 3.0 mm) for 2.5 s, while in the dilating condition, the artificial pupil diameter was dilated by 140% (from 5.0 mm to 7.0 mm) for 2.5 s^10^.

### Apparatus

The experimental stimuli were presented on a 23-inch LCD monitor (EIZO FlexScan EV2451, 1920 × 1080 pixel resolution, refresh rate of 60 Hz) using PsychoPy 3.0. The infants sat on their parent’s lap approximately 40 cm from the screen and eye tracker (Tobii Pro Spectrum; Tobii Technology, Inc., Danderyd, Sweden), which was employed to record their eye movements. A Tobii Pro spectrum with freedom of head movement within an area of 34 cm × 26 cm × 65 cm was utilized. The gaze was recorded at 150 Hz, and a five-point calibration was conducted before beginning the experiment, with the successful calibration of all points being mandatory.

A camera (Logicool C920R) was set below the display to monitor and record the infant’s behavior while looking at the stimuli. An experimenter observed the infant’s behavior using a monitor connected to a camera.

Room lighting was kept consistent within and between subjects.

### Procedure

Infants’ pupillary responses to changes in the pupils of unfamiliar-race faces were measured using an eye tracker. The experiment was designed to measure two conditions: face orientation (upright or inverted) as the between-subjects factor and pupil diameter change (dilating or constricting) as the within-subject factor. To attract the infants’ attention to the monitor, an object motion (1.0 s–3.0 s) was presented on a grey background. The experimenter initiated the trial as soon as the infant began to be attentive toward the object’s motion. A fixation point of a small black cross (2.38° × 2.38°) was presented on a grey background for 1.0 s before presenting each stimulus. In the upright condition, a static pupil (0.5 s), followed by a changing (dilating/constricting) pupil (2.5 s) for each face (six female faces of unfamiliar-races), was presented for 24 trials. The presentation sequence was pseudo-randomized. No more than two consecutive trials were conducted for the same type of face and pupil-diameter changes (dilating or constricting). The entire experiment took approximately 2.5 min. In the inverted condition, all faces were presented upside down.

### Data analysis

We calculated the mean pupil-diameter response from the stimulus onset (0 s) to the stimulus offset (3.0 s). The mean pupil diameter response was calculated as the average across pupil sizes from 0 to 3.0 s. Data files exported from the eye tracker were examined using MATLAB R2019a (MathWorks, Natick, MA, USA). We excluded individual trials that were missing more than 50% of the data from 1.0 s before stimulus onset (− 1.0 s) to stimulus offset (3.0 s), owing to inattention or technical problems. Gaps in the data for more than 15 samples were considered missing; however, smaller gaps were interpolated linearly. The data included gaps in under 16 samples, which were smoothed using a moving average over five samples. The baseline was the average of the pupil diameters, ranging from − 1.0 s to 0 s. This baseline was subtracted from the pupil diameter from − 1.0 s to 3.0 s. The mean pupil diameter was defined as the average pupil diameter from 0 s to 3.0 s.

The mean pupil diameters were analyzed using a two-way analysis of variance (ANOVA) with face orientation (upright or inverted) as a between-subject factor and pupil-change direction (dilating or constricting) as a within-subject factor.

Attritionary, we applied GLMM to analyze the data^30–32^. GLMM is an extension of the ordinary general linear model, which allows the analysis of clustered categorical data. We used the function glmer in the R^29^ (version 4.0.2) package lme4^34^ (version 1.1.26) for fitting GLMM. We initially included pupil change direction (dilating or constricting), face orientation (upright or inverted), age (5–6-month-old or 7–8-month-old), and all their interactions as fixed effects, and trial and individual differences as random effects.

### Supplementary Information

Supplementary Information.

## Data Availability

Raw data are available at Mendeley Data: https://doi.org/10.17632/4r535h77cp.1
