# Supplementary material for: The other-race effect of pupil contagion in infancy
Source: Sci Rep. 2024 Apr 24;14:9418. doi: 10.1038/s41598-024-59937-0 (PMC11043439; doi:10.1038/s41598-024-59937-0)
Supplement: Supplementary file 1 — Supplementary Information. [file 41598_2024_59937_MOESM1_ESM.docx]

Supplementary Material

# Results of GLMM

We applied GLMM to analyze the pupil response data (Agresti, 2007; Bolker et al., 2009; Moscatelli et al., 2012). GLMM is an extension of the ordinary general linear model, which allows the analysis of clustered categorical data. We used the function lmer in the R (version 4.0.2; R Core Team, 2020) package lme4 (version 1.1.26) (Bates et al., 2015) for fitting GLMM. We initially included the following fixed effects in the model: pupil change direction (dilating or constricting), face orientation (upright or inverted), age (5–6-month old or 7–8-month old), and all their interactions. Common across all models was that pupil-diameter change from baseline in individual trials was the dependent variable and that there were random intercepts for participants and trials. To obtain the most parsimonious model with the best fit, non-significant effects were removed one at a time, starting with the higher-order interactions. Via likelihood ratio tests, we verified whether the removal of a non-significant factor improved the fit of the model or not, in accordance with the most standard model-selection procedure. The result of model selection showed that the model with pupil change direction as fixed effect is most parsimonious model with the best fit (**Table 1**).

**Table1 The result of model selection**

| Backward reduced random effect | | | | | | | |
| --- | --- | --- | --- | --- | --- | --- | --- |
|  | | **Log likelihood** | | | **LRT** | | **p** |
| none | | 214509 | | |  | |  |
| 1 + pupil change direction * face orientation * age \| participants | | 214507 | | | 4.2 | | 0.83 |
| pupil change direction + presentation area + age + pupil change direction : face orientation + pupil change direction : age + face orientation : age \| participants | | 214507 | | | 0.2 | | 1.00 |
| pupil change direction + presentation area + age + pupil change direction : age + face orientation : age \| participants | | 214506 | | | 2.0 | | 0.92 |
| pupil change direction + presentation area + age + pupil change direction : age \| participants | | 214504 | | | 3.3 | | 0.65 |
| 1 + pupil change direction * face orientation * age \|trials | | 214100 | | | 807.7 | | < 0.001 |
| pupil change direction + age + pupil change direction : age \|participants | | 209839 | | | 9329.7 | | < 0.001 |
|  | |  | | |  | |  |
| Backward reduced fixed effect | |  | | |  | |  |
|  | **F** | | **Num df** | **Den df** | | **p** | |
| pupil change direction : presentation area : age | 8.71 | | 1 | 54.8 | | 0.00464 | |

Note: Satterthwaite method for degrees of freedom

As shown in Table1, the best fit model included pupil change direction, face orientation, age, and all their interactions as fix effect, and by-participants random slope for pupil change direction, age, and interaction between pupil change direction and age, and by-trials random slope for pupil change direction, face orientation, age, and all their interactions and intercepts for trials. We applied GLMM to analyze the pupil response data using this model. The GLMM revealed a significant effect (pupil change direction: b = 0.0847, SE = 0.0182, t = 4.66, p < 0.001), a significant interaction between age and pupil change direction (b = -0.0714, SE = 0.0228, t = -3.14, p = 0.00297), and a three-way interaction between face orientation, age group, and pupil-change direction (b = 0.0883, SE = 0.0299, t = 2.95, p = 0.00464) (**Table 2**).

**Table2 The result of GLMM**

| Fixed effect parameter estimates | | | | | | | | | | | | | | |
| --- | --- | --- | --- | --- | --- | --- | --- | --- | --- | --- | --- | --- | --- | --- |
|  | | |  | |  | **95% Confidence Interval** | | | |  | | |  |  |
| **Name** | | **Estimate** | | **SE** | | **Lower** | | **Upper** | | **df** | | **t** | | **p** |
| (Intercept) | | | -0.114 | 0.0214 | | -0.157 | | -0.0711 | | 58.6 | | -5.37 | | < 0.001 |
| pupil change direction | | | 0.0847 | 0.0182 | | 0.0477 | | 0.122 | | 34.6 | | 4.66 | | < 0.001 |
| face orientation | | | -0.000915 | 0.0299 | | -0.0607 | | 0.0589 | | 58.0 | | -0.0310 | | 0.976 |
| age | | | 0.0220 | 0.0287 | | -0.354 | | -0.0794 | | 57.1 | | 0.769 | | 0.445 |
| pupil change direction : face orientation | | | 0.00414 | 0.0236 | | -0.0433 | | 0.0516 | | 43.6 | | 0.176 | | 0.861 |
| pupil change direction : age | | | -0.714 | 0.0228 | | -0.117 | | -0.0256 | | 46.6 | | -3.14 | | 0.00297 |
| face orientation : age | | | -0.00937 | 0.0410 | | -0.0915 | | 0.0727 | | 58.0 | | -0.228 | | 0.820 |
| pupil change direction : face orientation: age | | | 0.0883 | 0.0299 | | 0.0284 | | 0.148 | | 54.8 | | 2.95 | | 0.00464 |
|  | | | | | | | | | | | | | | |
| Random Components | | | | | | | | | | |  | | | |
| **Groups** | **Name** | | | | | | **SD** | | **Variance** | |  | | | |
| participants | (Intercept) | | | | | | 0.0980 | | 0.00960 | |  | | | |
|  | pupil change direction | | | | | | 0.0635 | | 0.00403 | |  | | | |
|  | Age | | | | | | 0.135 | | 0.0181 | |  | | | |
|  | pupil change direction : age | | | | | | 0.0892 | | 0.00795 | |  | | | |
| trials | (Intercept) | | | | | | 0.0296 | | 0.000879 | |  | | | |
|  | pupil change direction | | | | | | 0.0450 | | 0.00203 | |  | | | |
|  | face orientation | | | | | | 0.0387 | | 0.00150 | |  | | | |
|  | age | | | | | | 0.0339 | | 0.00115 | |  | | | |
|  | pupil change direction : face orientation | | | | | | 0.0526 | | 0.00277 | |  | | | |
|  | pupil change direction : age | | | | | | 0.0489 | | 0.00239 | |  | | | |
|  | face orientation : age | | | | | | 0.0528 | | 0.00279 | |  | | | |
|  | pupil change direction : face orientation: age | | | | | | 0.0554 | | 0.00307 | |  | | | |
| residual |  | | | | | | 0.173 | | 0.0299 | |  | | | |

Note: Number of Obs: 640311, Groups: participants, 50; trials 12
